# Supplementary material for: N-Glycopedia: Libraries for Native N-glycan Structural Analysis
Source: bioRxiv. 2025 Jun 10:2025.06.09.658590. Preprint. [Version 1] doi: 10.1101/2025.06.09.658590 (PMC12259029; doi:10.1101/2025.06.09.658590)
Supplement: Supplement 1 [file media-1.pdf]

N-Glycopedia: Libraries for Native *N*-glycan Structural Analysis

Christopher Ashwood[1,2,3], Richard D Cummings[1]

[1] Department of Surgery, Division of Surgical Sciences, Beth Israel Deaconess Medical Center, Harvard Medical School, Boston, Massachusetts 02215, United States

[2] Glycomics Core, Beth Israel Deaconess Medical Center, Harvard Medical School, Boston, Massachusetts 02215, United States

[3] Protea Glycosciences Pty Ltd., Wollongong, NSW 2500, Australia

Running Title: A library of N-glycan standards enables targeted glycomics

Table of Contents

- **Figure S1** All unique *N*-glycan structure standards analysed to generate *N*-glycopedia and subsequently inform a targeted MS method
- **Figure S2** Compozitor output for the 91 glycan compositions assayed in *N*-glycopedia
- **Figure S3** Free *N*-glycan peak width is inversely correlated to PGC column temperature
- **Figure S4** Vendor cross-validation for six *N*-glycan structures confirms structural purity and identity
- **Figure S5** Unscheduled SRM enables quantitation of glycans not covered by *N*-glycopedia
- **Figure S6** MS2 matching scores across different mass spectrometers improve with recalibrated collision energy

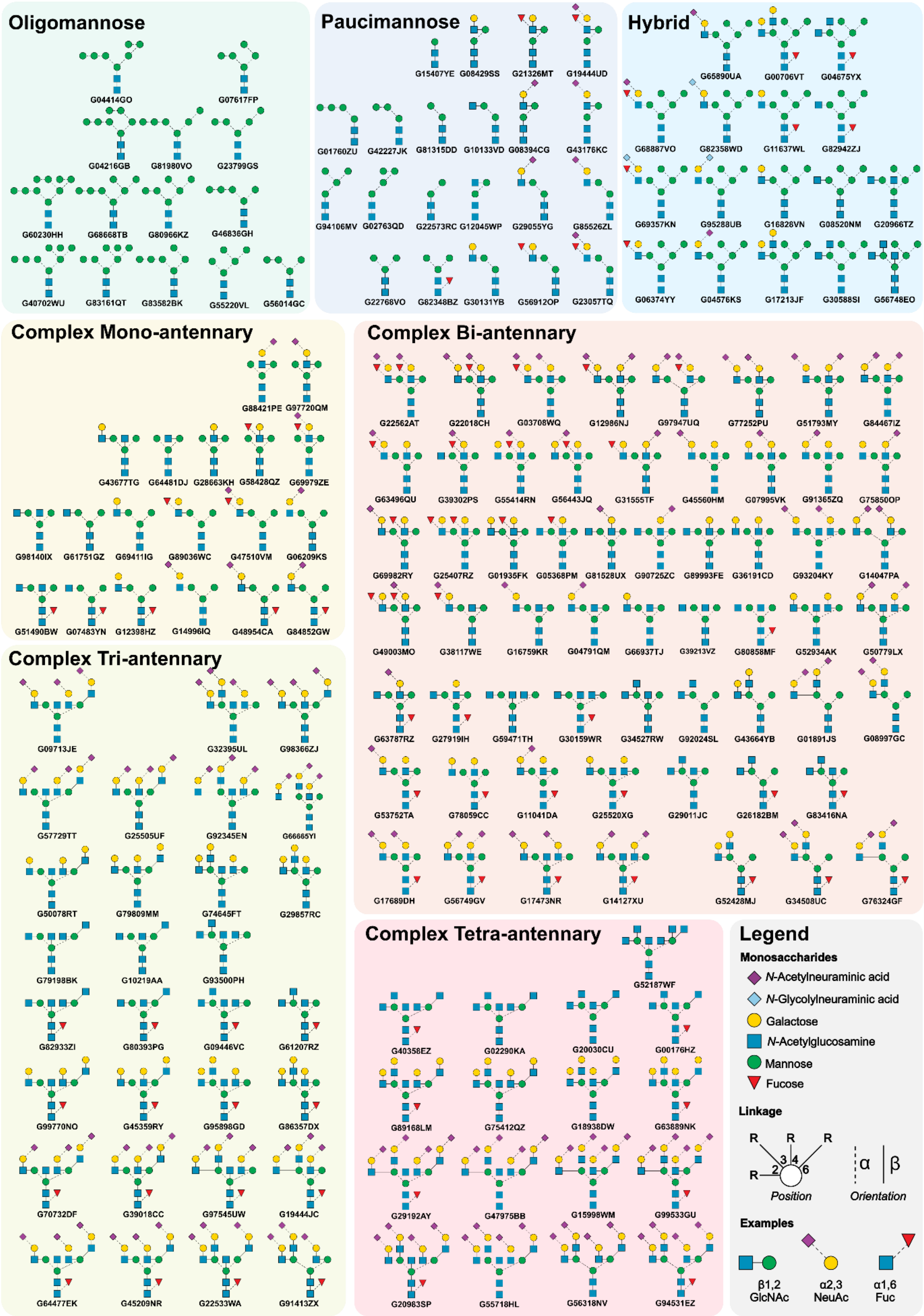

Figure S1 All unique *N*-glycan structure standards analysed to generate *N*-glycopedia and subsequently inform a targeted MS method

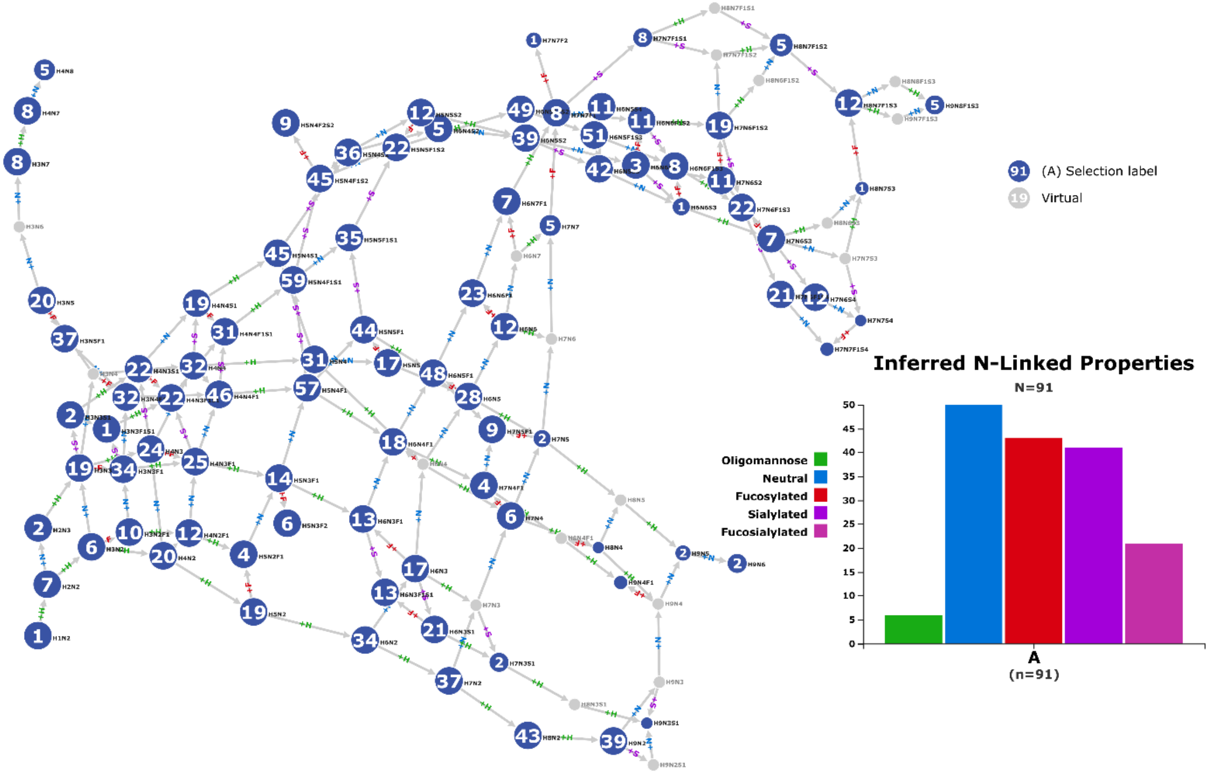

Figure S2 Compozitor output for the 91 glycan compositions assayed in *N*-glycopedia

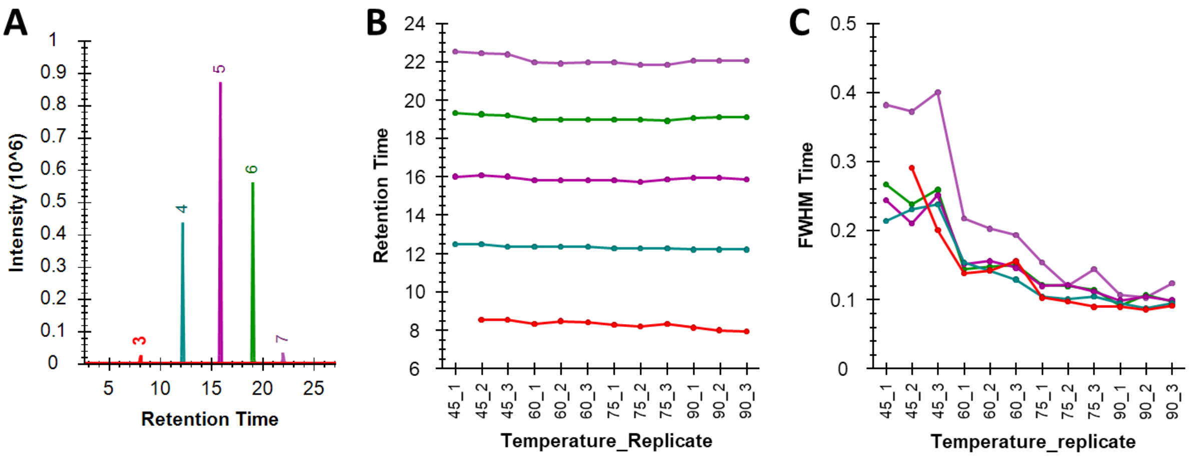

Figure S3 Free *N*-glycan peak width is inversely correlated to PGC column temperature

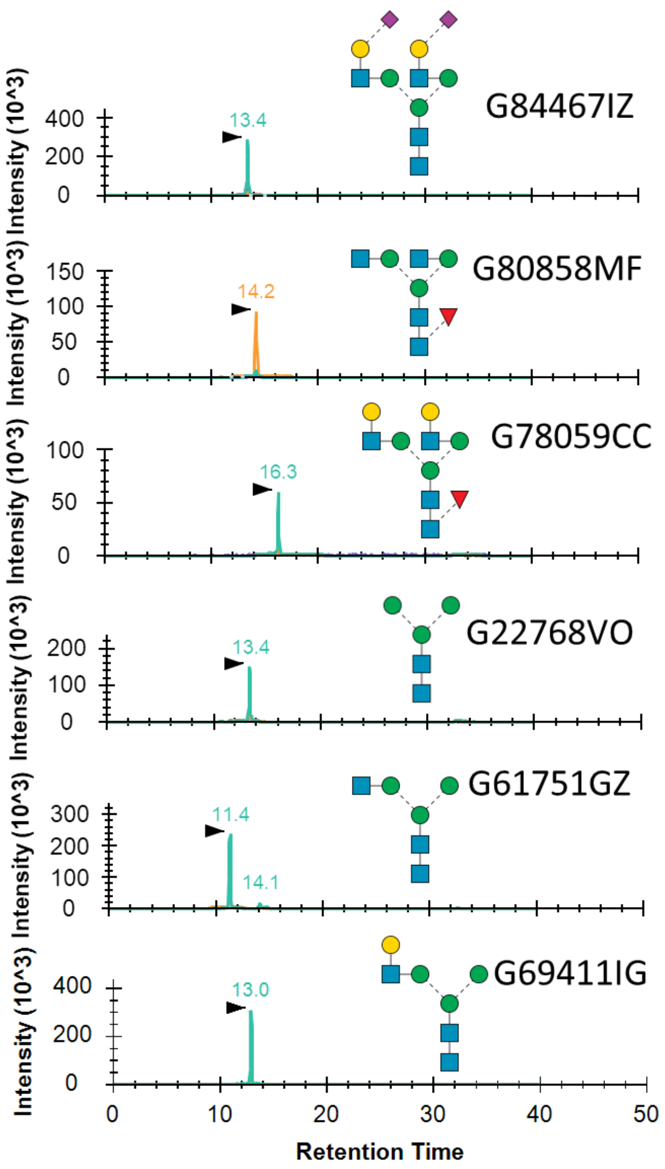

Figure S4 Vendor cross-validation for six *N*-glycan structures confirms structural purity and identity

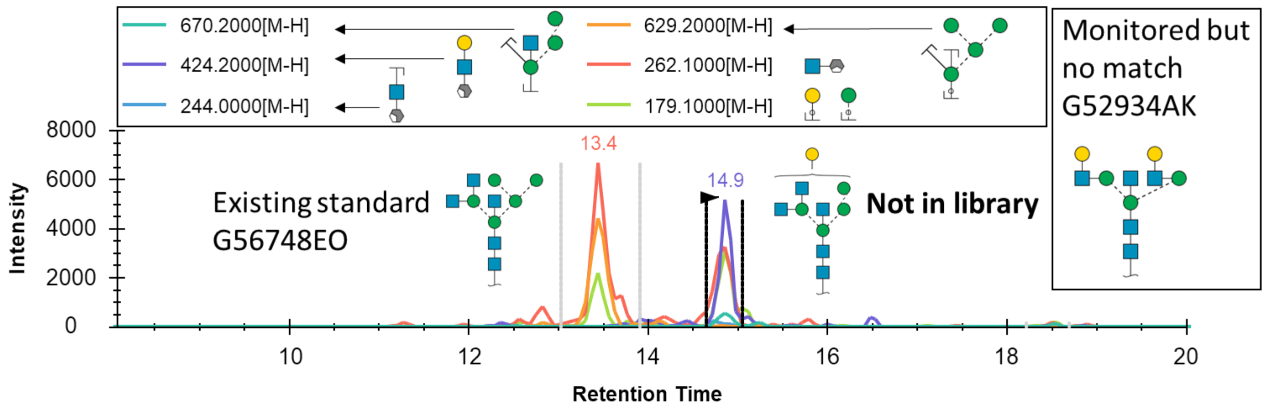

Figure S5 Unscheduled SRM enables quantitation of glycans not covered by *N*-glycopedia

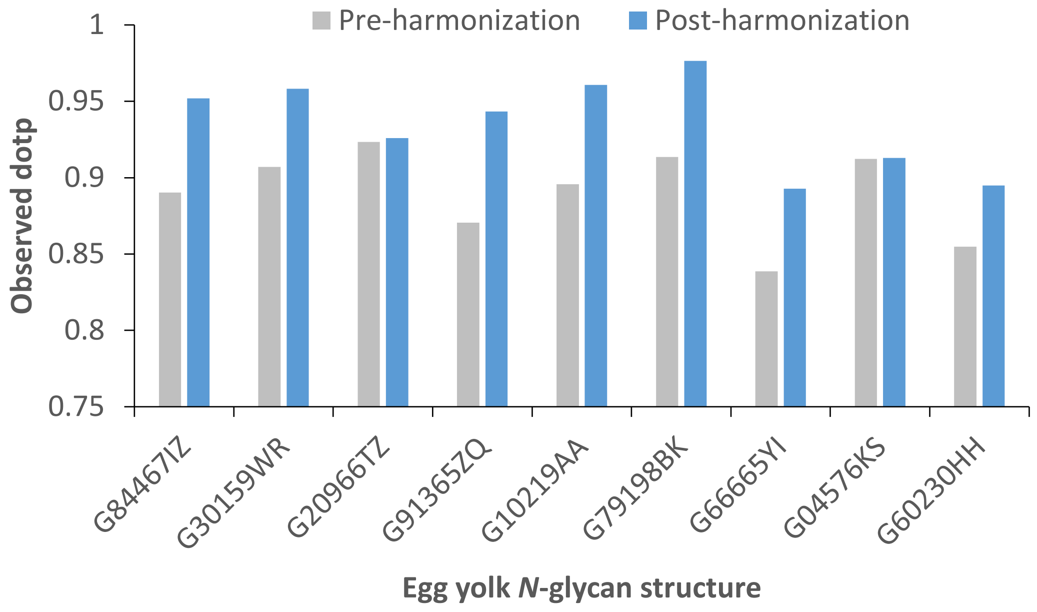

Figure S6 MS2 matching scores across different mass spectrometers improve with recalibrated collision energy
